# Droplet-Based Microfluidic Preparation of Shape-Variable Alginate Hydrogel Magnetic Micromotors

**DOI:** 10.3390/nano12010115

**Published:** 2021-12-30

**Authors:** Cheng Zhang, Yong Wang, Yuduo Chen, Xing Ma, Wenjun Chen

**Affiliations:** Sauvage Laboratory for Smart Materials, School of Materials Science and Engineering, Harbin Institute of Technology (Shenzhen), Shenzhen 518055, China; youzishaxiao@gmail.com (C.Z.); 18b954090@stu.hit.edu.cn (Y.W.); chenyuduo@stu.hit.edu.cn (Y.C.)

**Keywords:** droplet-based microfluidics, magnetic micromotors, hydrogel

## Abstract

This article introduces a facile droplet-based microfluidic method for the preparation of Fe_3_O_4_-incorporated alginate hydrogel magnetic micromotors with variable shapes. By using droplet-based microfluidics and water diffusion, monodisperse (quasi-)spherical microparticles of sodium alginate and Fe_3_O_4_ (Na-Alg/Fe_3_O_4_) are obtained. The diameter varies from 31.9 to 102.7 µm with the initial concentration of Na-Alginate in dispersed fluid ranging from 0.09 to 9 mg/mL. Calcium chloride (CaCl_2_) is used for gelation, immediately transforming Na-Alg/Fe_3_O_4_ microparticles into Ca-Alginate hydrogel microparticles incorporating Fe_3_O_4_ nanoparticles, i.e., Ca-Alg/Fe_3_O_4_ micromotors. Spherical, droplet-like, and worm-like shapes are yielded depending on the concentration of CaCl_2_, which is explained by crosslinking and anisotropic swelling during the gelation. The locomotion of Ca-Alg/Fe_3_O_4_ micromotors is activated by applying external magnetic fields. Under the rotating magnetic field (5 mT, 1–15 Hz), spherical Ca-Alg/Fe_3_O_4_ micromotors exhibit an average advancing velocity up to 158.2 ± 8.6 µm/s, whereas worm-like Ca-Alg/Fe_3_O_4_ micromotors could be rotated for potential advancing. Under the magnetic field gradient (3 T/m), droplet-like Ca-Alg/Fe_3_O_4_ micromotors are pulled forward with the average velocity of 70.7 ± 2.8 µm/s. This article provides an inspiring and timesaving approach for the preparation of shape-variable hydrogel micromotors without using complex patterns or sophisticated facilities, which holds potential for biomedical applications such as targeted drug delivery.

## 1. Introduction

Micromotors are microdevices capable of converting the energy from the environment into autonomous motion. Micromotors show promising aspects for applications in biomedical engineering and environmental remediation [1,2,3,4,5]. Magnetic micromotors are propelled by external magnetic fields. Compared with other types of micromotors, magnetic micromotors have advantages such as remote control, fuel-free, recyclability, etc. [6]. In addition, magnetic micromotors are demonstrated to be excellent candidates for long-term navigation [7]. Various methods have been reported for the preparation of magnetic micromotors with different shapes. Spherical magnetic micromotors can be prepared via the template-assisted method with sputtering or incorporating magnetic materials [8,9,10]. Helical magnetic micromotors can be prepared by two-photon polymerization lithography [11] and 3D printing [12]. Other shapes are produced by using a specific pattern [10,13]. However, these methods require sophisticated facilities and are usually time-consuming. Therefore, they are difficult to be replicated in normal laboratories. On the other hand, these micromotors are commonly composed of rigid materials which are not biodegradable in aqueous environments [14,15]. Thus, facile fabrication methods are in demand for magnetic micromotors using soft biodegradable materials.

Droplet-based microfluidics is widely used for the preparation of monodisperse microparticles. Recently, it attracted much researchers’ attention for the fabrication of magnetic micromotors due to its low-cost and easy-to-use platform. Typically, magnetic nanoparticles are incorporated within micromotors by means of photopolymerization or crosslinking. Both spherical [14,15,16,17,18,19] and helical [20] shapes can be produced. It allows fabricating micromotors with narrow size distribution, which is essential for biomedical applications such as drug delivery.

As for the material of micromotors, alginate is a natural polysaccharide extracted mainly from brown algae. Alginate hydrogel has been extensively used in drug delivery and tissue engineering because of its biocompatibility and biodegradability [21,22]. Besides, alginate hydrogel is easily synthesized through ionic crosslinking by divalent cations such as Ca^2+^.

Combining these two points mentioned above in terms of the choice of the preparing method and material, here we describe a facile droplet-based microfluidic method for the preparation of alginate hydrogel magnetic micromotors. First, a droplet-based microfluidic system is built for the generation of droplets of Na-alginate and Fe_3_O_4_ (Na-Alg/Fe_3_O_4_) in dimethyl carbonate (DMC). After water diffusion (details in Section 2.3), monodisperse (quasi-spherical) Na-Alg/Fe_3_O_4_ microparticles are obtained. An aqueous solution of calcium chloride (CaCl_2_) is used to achieve the gelation, transforming Na-Alg/Fe_3_O_4_ microparticles into Ca-Alginate hydrogel microparticles incorporating Fe_3_O_4_ nanoparticles (Ca-Alg/Fe_3_O_4_ micromotors). Spherical, droplet-like, and worm-like shapes are produced depending on the concentration of CaCl_2_, which avoids the use of complex patterns or sophisticated facilities. Besides, gelation being immediate makes the method timesaving. The mechanism of deformation is proposed based on the competition between crosslinking and anisotropic swelling during the gelation. Finally, both rotating magnetic field and magnetic field gradient are employed to activate the locomotion of Ca-Alg/Fe_3_O_4_ micromotors, showing their potentials for micro/nano-motors based drug delivery systems.

## 2. Materials and Methods

### 2.1. Chemical Materials

Na-Alginate (90%, M/G 1:2), DMC (99%), CaCl_2_ (96%), FeSO_4_·7H_2_O, fluorescein isothiocyanate isomer I (FITC, 90%) were all obtained from MACKLIN (Shanghai, China). FeCl_3_·6H_2_O, trisodium citrate dihydrate and NH_3_·H_2_O were obtained from BAISHI (Tianjin, China), DAMAO (Tianjin, China) and ALADDIN (Shanghai, China), respectively. All chemical materials are of analytical grade and used without further purification.

### 2.2. Synthesis of Fe_3_O_4_@Trisodium Citrate Nanoparticles

A total of 100 mL of DI water was heated in a three-neck flask at 80 °C for 20 min with the protection of nitrogen. An amount of 10 mL of FeCl_3_ solution (100 mg/mL), 5 mL of FeSO_4_ solution (100 mg/mL), 4.5 mL of NH_3_·H_2_O and 5 mL of trisodium citrate solution (400 mg/mL) were successively added into the flask. The mixture was blended at 1000 rpm for 90 min. Fe_3_O_4_@trisodium citrate nanoparticles (named for short as Fe_3_O_4_ nanoparticles thereafter) were collected by centrifugation (12,000× rpm, 10 min). Fe_3_O_4_ nanoparticles were cleaned 3 times with DI water.

### 2.3. Preparation of Na-Alg/Fe_3_O_4_ Microparticles via Droplet-Based Microfluidics and Water Diffusion

**Figure 1:**
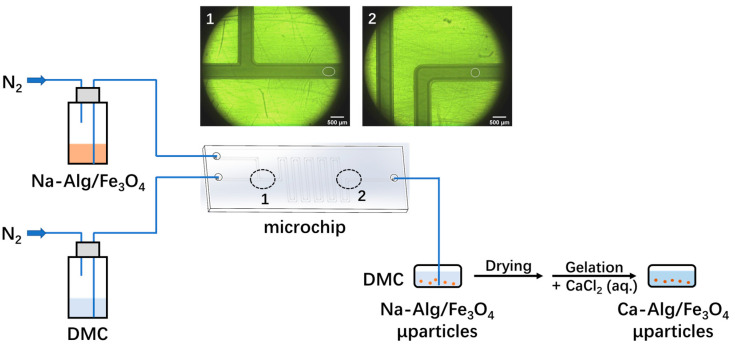
Schematic illustration of the preparation of Ca-Alg/Fe_3_O_4_ micromotors, with optical microscopic images of a droplet (outlined in white) generated right after the T-junction at Position 1 (insert microscopic image 1) and a droplet near the outlet of microchip at Position 2 (insert microscopic image 2).

A droplet-based microfluidic system was built by connecting a microchip (cubic channel, side length 500 µm) fabricated by 3D-printing with commercially available fluorinated ethylene propylene (FEP) capillary tubes (external diameter 1/16′′, internal diameter 500 µm) (Figure 1). The dispersed fluid was an aqueous solution of Na-Alginate (0.09, 0.9, 9 mg/mL) mixed with Fe_3_O_4_ nanoparticles (0.36 mg/mL), abbreviated as Na-Alg/Fe_3_O_4_. The continuous fluid was DMC. No surfactant was used because it created problems for further observation (Figure S1) and removal. With the pressure of nitrogen (50 mbar), the solution of Na-Alg/Fe_3_O_4_ and DMC were, respectively, injected into the microchip through two inlets. The flowrate of each fluid was controlled by a gas pump (ELVEFLOW). Due to the immiscibility between these two fluids, at the T-junction on chip (position 1 on the microchip in Figure 1), droplets of Na-Alg/Fe_3_O_4_ were generated in DMC, followed by droplet shrinkage. The principle of droplet shrinkage has been reported in our previous article [23]. Briefly, despite the immiscibility between DMC and water, DMC has a low solubility of water (3 wt%) [24]. Thus, after the droplet generation, water diffused gradually from each droplet to DMC, causing droplet shrinkage. It was observed by comparing the droplet at the T-junction (position 1 on chip in Figure 1) and that near the outlet of microchip (position 2 on chip in Figure 1). This process is called on-chip droplet shrinkage to indicate that it happens in the microchip. Droplets were collected with the yield of 1 droplet/s in a glass Petri dish filled with DMC through a capillary tube (length 46 cm). Note that the yield varies with experimental parameters, such as the gas pressure, the microchannel dimension, etc. Droplet shrinkage continued out of the microchip (off-chip droplet shrinkage) until the equilibrium was reached. Finally, Na-Alg/Fe_3_O_4_ microparticles were obtained.

Note that the serpentine channel in the microchip was specially designed to prolong the residence time of droplet ($\tau_{droplet}$). With the straight channel, $\tau_{droplet}$ was relatively short. On-chip droplet shrinkage was not efficient. Large droplets were observed at the outlet of microchip. Besides, they were likely to stagnate there where a capillary tube was connected, probably due to the imperfect connection or a minor flaw in the capillary tube. With water diffusion, the stagnating droplets became condensed and at last clogged the channel. By using the serpentine channel, $\tau_{droplet}$ was prolonged. It allowed for an efficient on-chip droplet shrinkage. Droplets were smaller at the outlet of microchip. Therefore, they were less likely to stagnate or eventually cause clogging there. Moreover, with an elevated droplet shrinkage, the distance between two droplets was also prolonged. At the end of the capillary tube in the glass Petri dish, there was less tendency for droplet coalescence, which was advantageous for preparing monodisperse microparticles.

### 2.4. Preparation of Ca-Alg/Fe_3_O_4_ Micromotors via Ionic Crosslinking

Na-Alg/Fe_3_O_4_ microparticles were dried in air with the evaporation of DMC. An aqueous solution of CaCl_2_ (10, 1, 0.1, 0.01 wt%) was added, transforming Na-Alginate into Ca-Alginate hydrogel via ionic crosslinking. Fe_3_O_4_ nanoparticles were incorporated inside. The ensemble was used as the hydrogel magnetic micromotor and named for short as Ca-Alg/Fe_3_O_4_ micromotor thereafter. It should be mentioned that the gelation process is immediate. Thus, the production of Ca-Alg/Fe_3_O_4_ micromotors mainly depends on the microfluidic experimental parameters and the quantity of micromotors required.

### 2.5. Characterization of Na-Alg/Fe_3_O_4_ Microparticles and Ca-Alg/Fe_3_O_4_ Micromotors

#### 2.5.1. Scanning Electron Microscope

Scanning electron microscope (SEM, Phenom ProX, Phenom-World, The Netherlands) was used to characterize the morphology of Na-Alg/Fe_3_O_4_ microparticles and Ca-Alg/Fe_3_O_4_ micromotors. The SEM sample was prepared by adhering dried particles to a copper sample-holder with a conductive tape. Some particles were broken after this step, which revealed information for the interior structure [23]. For a better resolution, all samples were coated by a thin layer of gold (Au) before SEM observation. The acceleration voltage of SEM was 15 kV. Energy-dispersive X-ray (EDX, Phenom) mapping analysis was conducted for element detection.

#### 2.5.2. Confocal Laser Scanning Microscope

Confocal laser scanning microscope (CLSM, Nikon A1, Nikon Company Ltd., Tokyo, Japan) was used to characterize the distribution of Fe_3_O_4_ nanoparticles inside Ca-Alg/Fe_3_O_4_ micromotors. For this purpose, FITC was initially conjugated with Fe_3_O_4_ nanoparticles by surface modification to form FITC-labeled Fe_3_O_4_ nanoparticles. They were then mixed with the solution of Na-alginate. After the protocol described above for the preparation of Ca-Alg/Fe_3_O_4_ micromotors, samples were immerged in DI water and observed by CLSM. The distribution of FITC represented that of Fe_3_O_4_ nanoparticles inside Ca-Alg/Fe_3_O_4_ micromotors. The 3D-image reconstruction was carried out to characterize the spatial distribution of Fe_3_O_4_ nanoparticles.

#### 2.5.3. Locomotion of Ca-Alg/Fe_3_O_4_ Micromotors in External Magnetic Fields

A home-made magnetic field generator was constructed [25]. It consisted of electromagnetic coils, a function generator (FY8300S) and a power amplifier (HSLFSun GLY-FP1000, Lab.Gruppen, Kungsbacka, Sweden). It allowed control of the magnetic field strength and orientation in the X, Y and Z axes by modulating the current in each electromagnetic coil. Sinusoidal function with a phase difference of 90° was used to generate the rotating magnetic field whose field vector varied regularly around the X axis in the Y-Z plane. Only one electromagnetic coil was activated to produce the magnetic field gradient at X axis. A glass slide was placed in the center of electromagnetic coils. A total of 20 µL of suspension consisting of Ca-Alg/Fe_3_O_4_ micromotors and water was added on the glass slide. Upon application of the external magnetic field, the movement of Ca-Alg/Fe_3_O_4_ micromotors was observed by an optical microscope (Leica DMi8, Leica Microsystems, Wetzlar, Germany) coupled with a CCD camera. The movement video was recorded and analyzed to calculate the advancing velocity of Ca-Alg/Fe_3_O_4_ micromotors.

## 3. Results and Discussion

### 3.1. Na-Alg/Fe_3_O_4_ Microparticles

Droplet-based microfluidic experiments were performed with three different initial concentrations of Na-Alginate in dispersed fluid ($C_{i,Na\text{-}Alg}$): 0.09, 0.9 and 9 mg/mL. The concentration of Fe_3_O_4_ ($C_{i,Fe_{3}O_{4}}$) was fixed at 0.36 mg/mL for all experiments.

#### 3.1.1. Diameter and Volume

**Figure 2:**
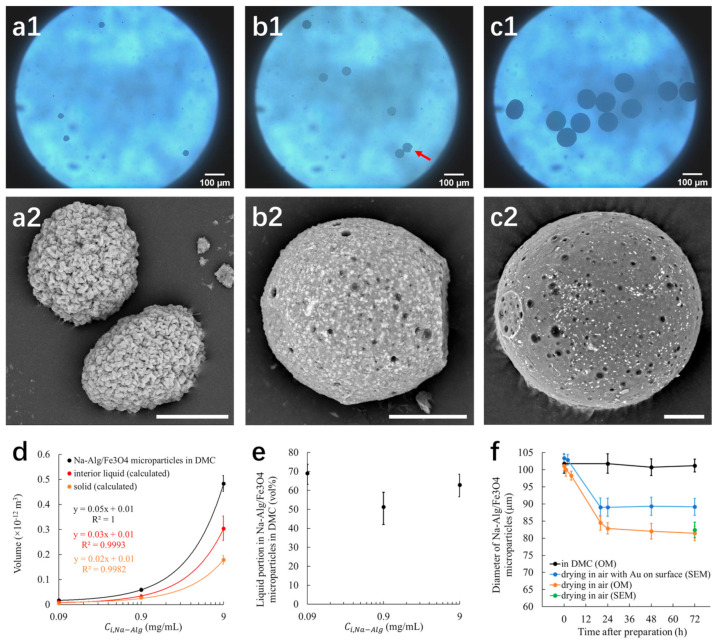
(**a1**,**b1**,**c1**) Optical microscopic images and (**a2**,**b2**,**c2**) SEM images of Na-Alg/Fe_3_O_4_ microparticles. The initial concentration of Na-Alginate in dispersed fluid ($C_{i,Na\text{-}Alg}$ ) for the preparation is (**a1**,**a2**) 0.09 mg/mL, (**b1**,**b2**) 0.9 mg/mL, (**c1**,**c2)** 9 mg/mL. The red arrow in b1 shows the neat cross section. SEM scale bar 20 µm. Relationship between $C_{i,Na\text{-}Alg}$ and (**d**) Na-Alg/Fe_3_O_4_ microparticle volume, interior liquid volume, solid volume; (**e**) liquid portion in Na-Alg/Fe_3_O_4_ microparticles in DMC. (**f**) Evolution of the diameter of Na-Alg/Fe_3_O_4_ microparticles (prepared with $C_{i,Na\text{-}Alg}$ at 9 mg/mL) in different media.

Monodisperse (quasi-)spherical Na-Alg/Fe_3_O_4_ microparticles were observed in DMC with an optical microscope (Figure 2(a1,c1)). The diameter of Na-Alg/Fe_3_O_4_ microparticles in DMC ($d_{particle,DMC}$) increases with $C_{i,Na\text{-}Alg}$ (Table 1). $d_{particle,DMC}$ varies from 31.9 to 102.7 µm with $C_{i,Na\text{-}Alg}$ ranging from 0.09 to 9 mg/mL.

The volume of Na-Alg/Fe_3_O_4_ microparticles in DMC ($V_{particle,DMC}$) is calculated according to the Equation (1).

(1) $$V_{particle,DMC}=\frac{4}{3}\pi {\left(\frac{d_{particle,DMC}}{2}\right)}^{3}$$

$V_{particle,DMC}$ increases with $C_{i,Na\text{-}Alg}$ (black symbols in Figure 2d), whose relationship is well fitted by a linear equation (written in black in Figure 2d). On the other hand, the diffusion of Na-Alginate and Fe_3_O_4_ from droplets to DMC is negligeable during the water diffusion [26]. Thus, the volume of Na-Alginate ($V_{Na\text{-}Alg}$) and the volume of Fe_3_O_4_ ($V_{Fe_{3}O_{4}}$) are the same as the corresponding volume in the initially generated droplet. $V_{Na\text{-}Alg}$ is calculated by dividing the mass of Na-Alginate ($m_{Na\text{-}Alg}$) with the density of Na-Alginate ($\rho_{Na\text{-}Alg}$). $m_{Na\text{-}Alg}$ is calculated by multiplying $C_{i,Na\text{-}Alg}$ with the initial droplet volume ($V_{i,droplet}$) (Equation (2)). $V_{Fe_{3}O_{4}}$ is calculated the same way (Equation (3)). Finally, the solid volume in the Na-Alg/Fe_3_O_4_ microparticle ($V_{solid}$), sum of $V_{Na\text{-}Alg}$ and $V_{Fe_{3}O_{4}}$ is calculated with the Equation (4).

(2) $$V_{particle,DMC}=\frac{4}{3}\pi {\left(\frac{d_{particle,DMC}}{2}\right)}^{3}$$

(3) $$V_{Fe_{3}O_{4}}=\frac{m_{Fe_{3}O_{4}}}{\rho_{Fe_{3}O_{4}}}=\frac{C_{i,Fe_{3}O_{4}}\times V_{i,droplet}}{\rho_{Fe_{3}O_{4}}}$$

(4) $$V_{Fe_{3}O_{4}}=\frac{m_{Fe_{3}O_{4}}}{\rho_{Fe_{3}O_{4}}}=\frac{C_{i,Fe_{3}O_{4}}\times V_{i,droplet}}{\rho_{Fe_{3}O_{4}}}$$

$V_{solid}$ increases with $C_{i,Na\text{-}Alg}$ (orange symbols in Figure 2d) with a leaner fitting equation (written in orange in Figure 2d). In fact, the linear relationship can be explained mathematically. In Equation (4), $\rho_{Na\text{-}Alg}$, $\rho_{Fe3O4}$ and $C_{i,Fe3O4}$ are constant. As for $V_{i,droplet}$, since it varies little from 0.3 to 0.4 µL for all experiments, it can also be considered as constant. $C_{i,Na\text{-}Alg}$ is the only variable. Consequently, Equation (4) gives a linear relationship for the function $V_{solid}=f\left(C_{i,Na\text{-}Alg}\right)$.

#### 3.1.2. Interior Liquid

It can be seen from Figure 2d that there is an evident gap between $V_{particle,DMC}$ and $V_{solid}$, indicating that the Na-Alg/Fe_3_O_4_ microparticle is not composed of condensed solid materials. In fact, flattened broken microparticles have been observed by SEM (Figure S2), which confirms the porous interior structure. For Na-Alg/Fe_3_O_4_ microparticles collected in DMC, there can be water trapped inside after water diffusion reaches equilibrium. DMC can also diffuse into Na-Alg/Fe_3_O_4_ microparticles. Thus, the difference between $V_{particle,DMC}$ and $V_{solid}$ is caused by the interior liquid which is water and DMC. The volume of the interior liquid ($V_{interior\;liquid}$) is simply calculated with Equation (5) and plotted with $C_{i,Na\text{-}Alg}$ in Figure 2d (red symbols). $V_{interior\;liquid}$ shows a linear relationship with $C_{i,Na\text{-}Alg}$ (fitting equation written in red in Figure 2d). The liquid portion, defined as the liquid volume percentage in the Na-Alg/Fe_3_O_4_ microparticle ($V_{interior\;liquid}\%$), is calculated by Equation (6). It is found that about 50–70% of the Na-Alg/Fe_3_O_4_ microparticle is filled with liquid, despite $C_{i,Na\text{-}Alg}$ used (Figure 2e).

(5) $$V_{interior\;liquid}=V_{particle,DMC}-V_{solid}$$

(6) $$V_{interior\;liquid}\%=\frac{V_{interior\;liquid}}{V_{particle,DMC}}\times 100\%$$

#### 3.1.3. Stability

Na-Alg/Fe_3_O_4_ microparticles were stored in DMC and air (25 °C, humidity 40%), respectively, to assess the stability in terms of diameter in 72 h after the preparation. Na-Alg/Fe_3_O_4_ microparticles were observed by both optical microscope and SEM. The diameter of Na-Alg/Fe_3_O_4_ microparticles is measured in three different ways. (1) In DMC with optical microscopic observation: the diameter is stable around 100 µm (black symbols in Figure 2f). (2) In air with optical microscopic observation: the diameter reduces from 100 to 82 µm within the first 24 h and stays unchanged for the following 48 h (orange symbols in Figure 2f and Figure S3(a1–a5)). It can be explained by the evaporation of the interior liquid in air. After being dried in air for 72 h, the same sample is also observed by SEM (Figure S4). The diameter measured from SEM observation (green symbol in Figure 2f) shows no evident difference from that from optical microscopic observation, indicating that the observing tool does not contribute to the difference of measurement. (3) Samples are coated by a thin layer of Au once and observed by SEM. They are stored in air each time after observation. The diameter reduces from 100 to 89 µm within the first 24 h and stays unchanged for the following 48 h (blue symbols in Figure 2f and Figure S3(a1–a5)). The final diameter is higher than that observed in air. It is due to the presence of deposited Au layer on the surface of Na-Alg/Fe_3_O_4_ microparticles which hinders the evaporation of the interior liquid. Consequently, larger microparticles are observed.

Note that with the same $C_{i,Na\text{-}Alg}$ at 9 mg/mL, $V_{solid}$ (Figure 2d) gives an equivalent diameter of 70 ± 2 µm. It is still lower than the last diameter measured in air with optical microscope (82 µm). Thus, we assume that the interior liquid is not all evaporated.

#### 3.1.4. Morphology

The morphology of Na-Alg/Fe_3_O_4_ microparticles was characterized by SEM. Different morphologies were observed depending on $C_{i,Na\text{-}Alg}$ used for the preparation. When $C_{i,Na\text{-}Alg}$ is extremely low at 0.09 mg/mL, the surface of Na-Alg/Fe_3_O_4_ microparticles is rough (Figure 2(a2)). By increasing $C_{i,Na\text{-}Alg}$ to 0.9 mg/mL, the obtained Na-Alg/Fe_3_O_4_ microparticles have a smooth and porous surface with a neat cross section (Figure 2(c2) and Figure S5(a1–a3)). The fact that this structure is also observed by optical microscope for Na-Alg/Fe_3_O_4_ microparticles in DMC (red arrow in Figure 2(b1)), demonstrating that it is not caused by vacuum during SEM observation. For the highest $C_{i,Na\text{-}Alg}$ at 9 mg/mL, Na-Alg/Fe_3_O_4_ microparticles have a smooth and porous surface with a tiny flat round trace (Figure 2(c2) and Figure S5(b1–b3)).

Since $C_{i,Fe_{3}O_{4}}$ and $V_{i,droplet}$ are constant parameters for all experiments, the quantity of Fe_3_O_4_ is fixed in Na-Alg/Fe_3_O_4_ microparticles. Thus, the difference of morphology is caused by the quantity of Na-Alginate. Based on the results, the mechanism of the formation of Na-Alg/Fe_3_O_4_ microparticles is proposed as follows.

#### 3.1.5. Mechanism of the Formation of Na-Alg/Fe_3_O_4_ Microparticles

**Figure 3:**
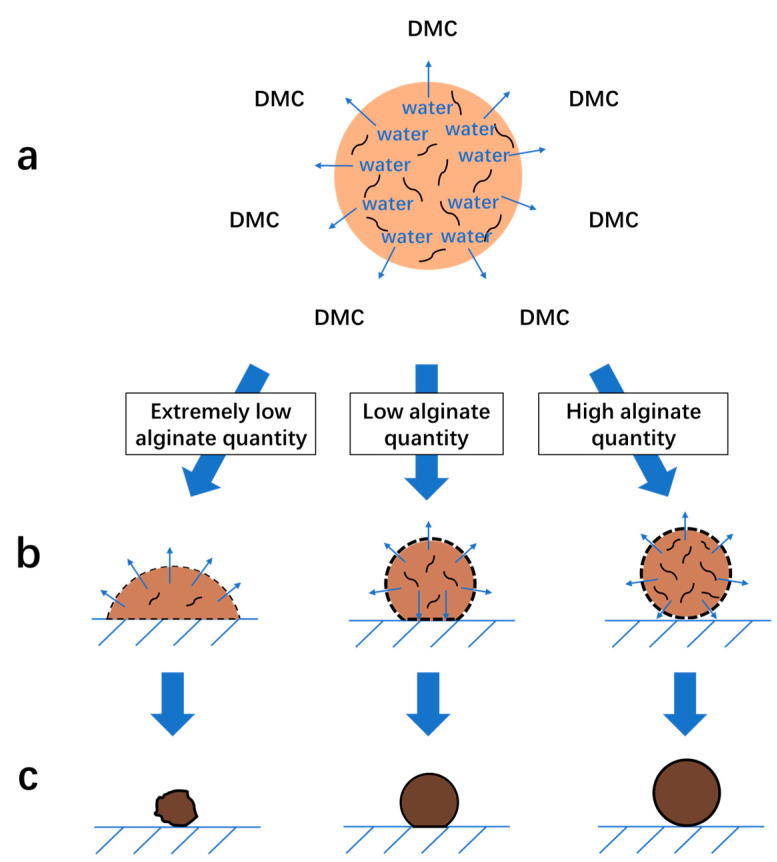
Illustration of the mechanism of the formation of Na-Alg/Fe_3_O_4_ microparticles in a glass Petri dish filled with DMC: (**a**) droplets fall with water diffusion; (**b**) droplets land on the glass bottom with a primary shell formed on the surface; (**c**) water diffusion reaches equilibrium and final microparticles are formed.

Na-Alg/Fe_3_O_4_ droplets are collected in DMC in a glass Petri dish. As droplets fall in DMC, water diffuses from droplets into DMC though the interface (Figure 3a). The diffusion of water brings about the migration of alginate polymeric chains until the interface [27]. Progressively, a primary porous shell structure is formed. When a droplet lands on the glass bottom, the deformation depends on the resistance of the shell which is influenced by the alginate quantity ($Q_{alginate}$) in the droplet (Figure 3b). When $Q_{alginate}$ is extremely low (corresponding to $C_{i,Na\text{-}Alg}$ = 0.09 mg/mL), the shell is not resistant enough to the shock upon landing, producing a spreading form. Since there is no sufficient alginate to form a full shell, the final structure is rather a random stock of Fe_3_O_4_ nanoparticles which is the major solid material in this case (Figure 3c). When $Q_{alginate}$ is increased but still low (corresponding to $C_{i,Na\text{-}Alg}$ = 0.9 mg/mL), the shell resists better the shock of glass. The droplet stays spherical with a neat cross section due to the contact with the glass, which explains the morphology observed by SEM. With the highest $Q_{alginate}$ (corresponding to $C_{i,Na\text{-}Alg}$ = 9 mg/mL), the shell is so strong that the droplet is hardly deformed. In the end, spherical microparticles are obtained with a tiny flat round trace left when touching the glass.

### 3.2. Ca-Alg/Fe_3_O_4_ Micromotors

An aqueous solution of CaCl_2_ (10, 1, 0.1, 0.01 wt%) was added to dried Na-Alg/Fe_3_O_4_ microparticles, transforming Na-Alg/Fe_3_O_4_ microparticles into Ca-Alg/Fe_3_O_4_ micromotors. Spherical, droplet-like, and worm-like shapes are obtained (Table 2). The influence of the concentration of CaCl_2_ ($C_{CaCl_{2}}$) is studied.

#### 3.2.1. Influence of $C_{CaCl_{2}}$ on the Shape of Ca-Alg/Fe_3_O_4_ Micromotors

(1) For Na-Alg/Fe_3_O_4_ microparticles prepared with $C_{i,Na\text{-}Alg}$ at 0.09 mg/mL

$Q_{alginate}$ is low in Na-Alg/Fe_3_O_4_ microparticles. The gelation does not produce any evident deformation with $C_{CaCl_{2}}$ varying from 0.1 to 10 wt%. Ca-Alg/Fe_3_O_4_ micromotors stay quasi-spherical. When the solution of CaCl_2_ is too diluted ($C_{CaCl_{2}}$ = 0.01 wt%), Na-Alg/Fe_3_O_4_ microparticles are almost dissolved.

(2) For Na-Alg/Fe_3_O_4_ microparticles prepared with $C_{i,Na\text{-}Alg}$ at 0.9 and 9 mg/mL

These Na-Alg/Fe_3_O_4_ microparticles have a similar manner of deformation depending on $C_{CaCl_{2}}$. With a concentrated solution of CaCl_2_ ($C_{CaCl_{2}}$= 10 wt%), no deformation is produced. Ca-Alg/Fe_3_O_4_ micromotors are spherical. When Na-Alg/Fe_3_O_4_ microparticles are immerged in a solution with $C_{CaCl_{2}}$ at 1 wt%, a tail is grown from the “defect” (neat cross section or tiny flat round trace, Figure 2(b2,c2)) on the surface of Na-Alg/Fe_3_O_4_ microparticles (Video S1). As a result, Ca-Alg/Fe_3_O_4_ micromotors have a droplet-like shape. By reducing $C_{CaCl_{2}}$ to 0.1 wt%, a longer tail is grown, creating the worm-like shape. Finally, the use of a solution with $C_{CaCl_{2}}$ at 0.01 wt% almost dissolves Na-Alg/Fe_3_O_4_ microparticles.

#### 3.2.2. Mechanism of the Deformation

When adding an aqueous solution of CaCl_2_ for the gelation, calcium cations diffuse into Na-Alg/Fe_3_O_4_ microparticles, causing ionic crosslinking. It allows strengthening bonds between alginate polymeric chains, which impedes further deformation. Meanwhile, water also diffuses into Na-Alg/Fe_3_O_4_ microparticles, causing swelling. The swelling is anisotropic because of the anisotropic morphology of Na-Alg/Fe_3_O_4_ microparticles with “defect” on the surface (neat cross section or tiny flat round trace, Figure 2(b2,c2)). Overall, in our case, the gelation process is a competition of crosslinking which impedes the deformation and anisotropic swelling which encourages the deformation.

When $C_{CaCl_{2}}$ is high at 10 wt%, calcium cations diffuse fast to Na-Alg/Fe_3_O_4_ microparticles to achieve crosslinking. The spherical shape is preserved with nearly no swelling. When $C_{CaCl_{2}}$ is 1 wt%, calcium cations diffuse more slowly to Na-Alg/Fe_3_O_4_ microparticles, leading to a slower crosslinking. It leaves time for anisotropic swelling. According to the result, the swelling is more important at the “defect” on the surface, which explains the growth of the tail. A further reduction in $C_{CaCl_{2}}$ to 0.1 wt% is more advantageous for anisotropic swelling, as there is more water and fewer calcium cations in the environment. A longer tail is grown. Furthermore, we note that with the anisotropic swelling, Na-Alg/Fe_3_O_4_ microparticle grow upwards from the glass bottom (Video S1) with a tail adhered to the glass. The tail bends when it can no longer bear the weight of the whole microparticle. It explains why certain tails are curved. Finally, when $C_{CaCl_{2}}$ is too low at 0.01 wt%, there are no sufficient calcium cations for crosslinking. Anisotropic swelling takes over crosslinking totally. Na-Alg/Fe_3_O_4_ microparticles are almost dissolved.

#### 3.2.3. Characterization of Fe_3_O_4_ Nanoparticles inside Ca-Alg/Fe_3_O_4_ Micromotors

**Figure 4:**
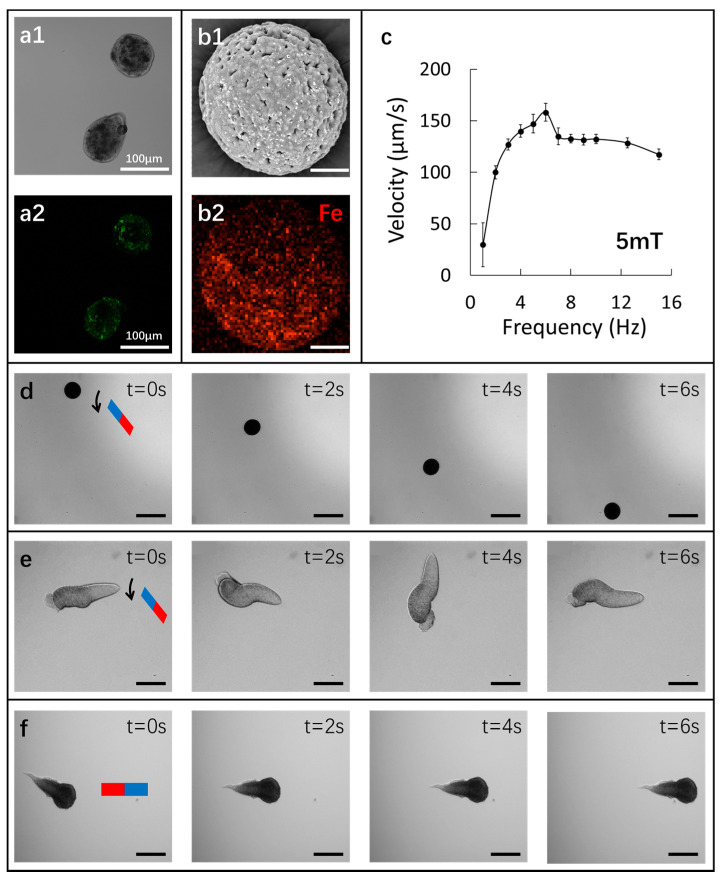
(**a1**) Bright-field and (**a2**) fluorescence microscopic image of micromotors with incorporated FITC-labeled Fe_3_O_4_ nanoparticles. (**b1**) SEM image and (**b2**) corresponding EDX mapping analysis of a Ca-Alg/Fe_3_O_4_ micromotor for iron (scale bar 20 µm). (**c**) Relationship between the advancing velocity of spherical micromotors (prepared with $C_{i,Na\text{-}Alg}$ at 9 mg/mL for the microfluidic experiment and $C_{CaCl_{2}}$ at 10 wt% for the gelation) and the frequency at 5 mT. Video snapshots (scale bar 200 µm) at different time intervals of a (**d**) spherical and (**e**) worm-like micromotor under the rotating magnetic field at 5 mT around X axis in Y-Z plane; (**f**) droplet-like micromotor under the X-axis magnetic field gradient of 3 T/m.

By using FITC-labeled Fe_3_O_4_ nanoparticles, the fabricated micromotors were observed by CLSM. The fluorescent image (Figure 4(a1,a2)) indicates that Fe_3_O_4_ nanoparticles are incorporated in Ca-Alginate hydrogel successfully. It is also verified by EDX mapping analysis (Figure 4(b1,b2)). Moreover, the 3D-image reconstruction by CLSM shows that Fe_3_O_4_ nanoparticles are mostly located at the bottom half semi-sphere of the micromotor (Video S2).

#### 3.2.4. Locomotion of Ca-Alg/Fe_3_O_4_ Micromotors

The locomotion of all three types of micromotors is always observed at the bottom of the glass Petri dish. For spherical and worm-like Ca-Alg/Fe_3_O_4_ micromotors, the locomotion is activated by applying a rotating magnetic field at 5 mT around X axis in Y-Z plane. The frequency (f) varies from 1 to 15 Hz. We observe that spherical micromotors move forward by rotation-enabled rolling (Figure 4d, Video S3). The advancing velocity ($v_{advancing}$) increases with f until the step-out frequency ($f_{step\text{-}out}$ = 6 Hz) is reached (Figure 4c). Then, $v_{advancing}$ declines with the frequency. The maximum $v_{advancing}$ is 158.2 ± 8.6 µm/s with f at 6 Hz. However, as for worm-like micromotors, with the application of rotating magnetic field, rotation behavior is observed, which can be further developed for advancing movement based on their helical structure (Figure 4e, Video S4).

With the symmetric structure, droplet-like Ca-Alg/Fe_3_O_4_ micromotors cannot move forward under the rotating magnetic field. The locomotion of a droplet-like Ca-Alg/Fe_3_O_4_ micromotor is activated by applying an X-axis magnetic field gradient. At 2 T/m, the micromotor is not actuated at all. At 3 T/m, a torque is first produced and turns the micromotor to align with the direction of the external magnetic field. Then, the micromotor is pulled forward (Figure 4f, Video S5) with an average velocity of 70.7 ± 2.8 µm/s.

Although all micromotors are not actuated in the same type of external magnetic field, we note that with the same current input in the electromagnetic coil, micromotors are more likely to be actuated by the rotating magnetic field than the magnetic field gradient. It can be explained by the fact that the friction force is generally higher when a micromotor is pulled forward than when it advances by rotation. Thus, the rotating magnetic field is preferably reported in the literature, whereas it demands a certain shape of micromotors, such as spherical and helical shapes. The present method allows producing the spherical shape. We hope that with further modification and precise control during gelation, the worm-like shape can be transformed into the well-defined shape such as helical structured motors.

## 4. Conclusions and Perspective

In conclusion, we present herein a facile droplet-based microfluidic preparation of Fe_3_O_4_-incorporated alginate hydrogel micromotors with variable shapes. First, monodisperse (quasi-)spherical Na-Alg/Fe_3_O_4_ microparticles are obtained by using droplet-based microfluidics and water diffusion. The diameter of Na-Alg/Fe_3_O_4_ microparticles varies from 31.9 to 102.7 µm, depending on the initial concentration of Na-Alginate in dispersed fluid. The mechanism of the formation of Na-Alg/Fe_3_O_4_ microparticles is proposed to explain different morphology observed by SEM. Second, an aqueous solution of CaCl_2_ is used for gelation, transforming Na-Alg/Fe_3_O_4_ microparticles into Ca-Alg/Fe_3_O_4_ micromotors. Spherical, droplet-like, and worm-like shapes are obtained, which is mainly affected by the concentration of CaCl_2_. The mechanism of deformation is proposed considering the crosslinking and anisotropic swelling during the gelation. Finally, Ca-Alg/Fe_3_O_4_ micromotors are actuated by applying external magnetic fields, showing their potential of developing functional micro/nano-motors.

This article provides an idea for producing different shapes of hydrogel micromotors without using complex patterns or sophisticated facilities. The method is timesaving and easy to be realized. In terms of the stability of micromotors, the size has been measured for 14 days consecutively and no evident change was found. Stored in pure water, micromotors demonstrated a decreasing locomotion probably due to the diffusion of Fe_3_O_4_ nanoparticles. Nevertheless, the advancing velocity of spherical micromotors was estimated to be stable within 2 days. For long-term utility or storage, work should be done to conserve the locomotion of micromotors. Future work will focus on integrating biomedical or biological agents, such as medical molecules and cells to achieve on-demand tasks such as drug delivery and cell transportation.

## Figures and Tables

**Table 1:** Diameter of Na-Alg/Fe_3_O_4_ microparticles in DMC ($d_{particle,DMC}$) in function of the initial concentration of Na-Alginate in dispersed fluid ($C_{i,Na\text{-}Alg}$).

$C_{i,Na\text{-}Alg}\;(\mathrm{mg}/\mathrm{mL})$	$d_{particle,DMC}\;(µm)$
0.09	31.9 ± 1.4
0.9	48.2 ± 1.6
9	102.7 ± 1.7

**Table 2:** Optical microscopic and schematic images of Ca-Alg/Fe_3_O_4_ micromotors with the indication of $C_{i,Na\text{-}Alg}$ and $C_{CaCl_{2}}$ for the preparation. Scale bar 100 µm.

$C_{CaCl_{2}}$	$C_{i,Na\text{-}Alg}=0.09\;\mathrm{mg}/\mathrm{mL}$	$C_{i,Na\text{-}Alg}=0.9\;\mathrm{mg}/\mathrm{mL}$	$C_{i,Na\text{-}Alg}=9\;\mathrm{mg}/\mathrm{mL}$
	Optical Schematic	Optical Schematic	Optical Schematic
**10 wt%**	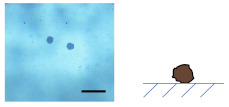	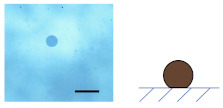	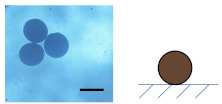
**1 wt%**	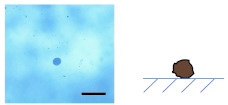	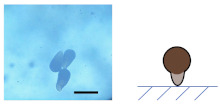	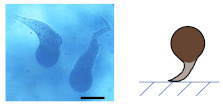
**0.1 wt%**	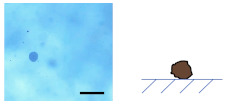	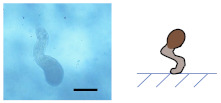	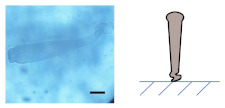
**0.01 wt%**	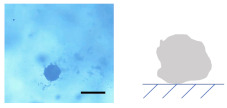	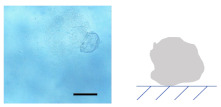	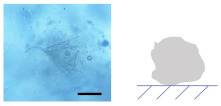

## Data Availability

Data can be available upon request from the authors.

## Supplementary Materials

The following supporting information can be downloaded at: https://www.mdpi.com/2079-4991/12/1/115/s1, Figure S1: SEM images of Na-Alg/Fe_3_O_4_ microparticles fabricated with the use of Tween 80 (surfactant) in DMC. $C_{i,Na\text{-}Alg}$ is 0.09 mg/mL (left) and 0.9 mg/mL (right) for the microfluidic experiment. Scale bar 10µm, Figure S2: SEM images of broken Na-Alg/Fe_3_O_4_ microparticles (prepared with $C_{i,Na\text{-}Alg}$ at 0.9 mg/mL for the microfluidic experiment). Scale bar 20 µm, Figure S3: (a1–a5) Optical microscopic images (in air) and (b1–b5) SEM images of the same Na-Alg/Fe_3_O_4_ microparticles (prepared with $C_{i,Na\text{-}Alg}$ at 9 mg/mL for the microfluidic experiment). From a1 to a5, also from b1 to b5, the observation time is t0 (just after the preparation), t0 + 20h, t0 + 24h, t0 + 48h, t0 + 72h. SEM scale bar 20 µm, Figure S4: SEM image of a Na-Alg/Fe_3_O_4_ microparticle stored in air for 72 h after preparation, Figure S5: SEM images of Na-Alg/Fe_3_O_4_ microparticles prepared with $C_{i,Na\text{-}Alg}$ at (a1–a3) 0.9 mg/mL and (b1–b3) 9 mg/mL for the microfluidic experiment. Scale bar 20 µm; Video S1: Adding CaCl_2_ ($C_{CaCl_{2}}$ = 0.1 wt%) to a dried Na-Alg/Fe_3_O_4_ microparticle (prepared with $C_{i,Na\text{-}Alg}$ = 0.9 mg/mL), Video S2: 3D-image reconstruction of Ca-Alg/Fe_3_O_4_ micromotors by CLSM. Ca-Alg/Fe_3_O_4_ micromotors were prepared with $C_{i,Na\text{-}Alg}$ = 1.4 mg/mL in dispersed fluid and $C_{CaCl_{2}}$ = 1 wt% for gelation, Video S3: Movement of a spherical Ca-Alg/Fe_3_O_4_ micromotor under the rotating magnetic field at 5 mT around X axis in Y-Z plane. The Ca-Alg/Fe_3_O_4_ micromotor was prepared with $C_{i,Na\text{-}Alg}$ at 9 mg/mL for the microfluidic experiment and $C_{CaCl_{2}}$ at 10 wt% for the gelation, Video S4: Movement of worm-like Ca-Alg/Fe_3_O_4_ micromotors under the rotating magnetic field at 5 mT around X axis in Y-Z plane. The Ca-Alg/Fe_3_O_4_ micromotors were prepared with $C_{i,Na\text{-}Alg}$ at 9 mg/mL for the microfluidic experiment and $C_{CaCl_{2}}$ at 0.1 wt% for the gelation, Video S5: Movement of a droplet-like Ca-Alg/Fe_3_O_4_ micromotor under the X-axis magnetic field gradient of 3 T/m. The Ca-Alg/Fe_3_O_4_ micromotor was prepared with $C_{i,Na\text{-}Alg}$ at 9 mg/mL for the microfluidic experiment and $C_{CaCl_{2}}$ at 1 wt% for the gelation.
