# Supplementary material for: Droplet-Based Microfluidic Preparation of Shape-Variable Alginate Hydrogel Magnetic Micromotors
Source: Nanomaterials (Basel). 2021 Dec 30;12(1):115. doi: 10.3390/nano12010115 (PMC8796028; doi:10.3390/nano12010115)
Supplement: Supplementary file 1 [file nanomaterials-12-00115-s001.zip › nanomaterials-1501305-supplementary.pdf]

Supplementary Materials

Droplet-Based Microfluidic Preparation of Shape-Variable Alginate Hydrogel Magnetic Micromotors

Cheng Zhang, Yong Wang, Yuduo Chen, Xing Ma * and Wenjun Chen *

Sauvage Laboratory for Smart Materials, School of Materials Science and Engineering, Harbin Institute of Technology (Shenzhen), Shenzhen 518055, China; youzishaxiao@gmail.com (C.Z.); 18b954090@stu.hit.edu.cn (Y.W.); chenyuduo@stu.hit.edu.cn (Y.C.)

* Correspondence: maxing@hit.edu.cn (X.M.); chenwenjun@hit.edu.cn (W.C.)

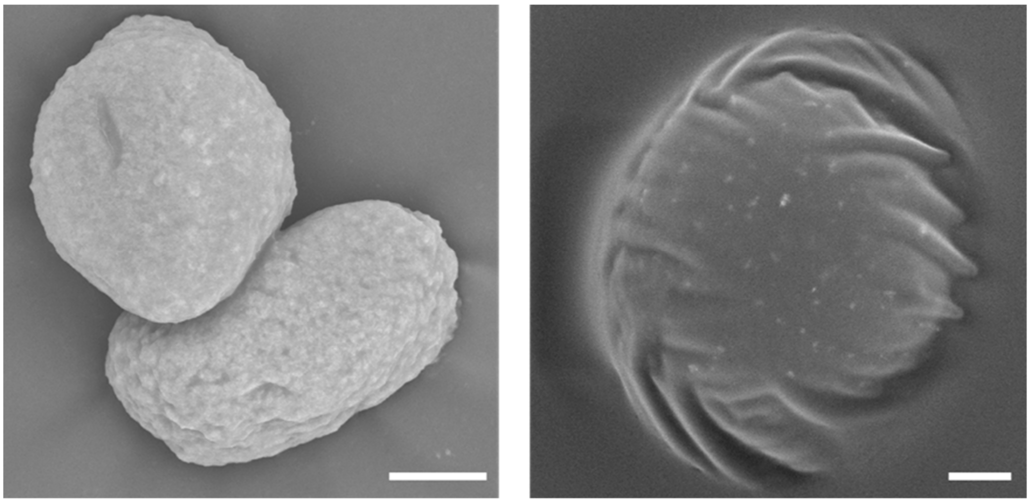

Figure S1. SEM images of Na-Alg/Fe_3O_4 microparticles fabricated with the use of Tween 80 (surfactant) in DMC. $C_{i,Na-Alg}$ is 0.09 mg/mL (left) and 0.9 mg/mL (right) for the microfluidic experiment. Scale bar 10μm.

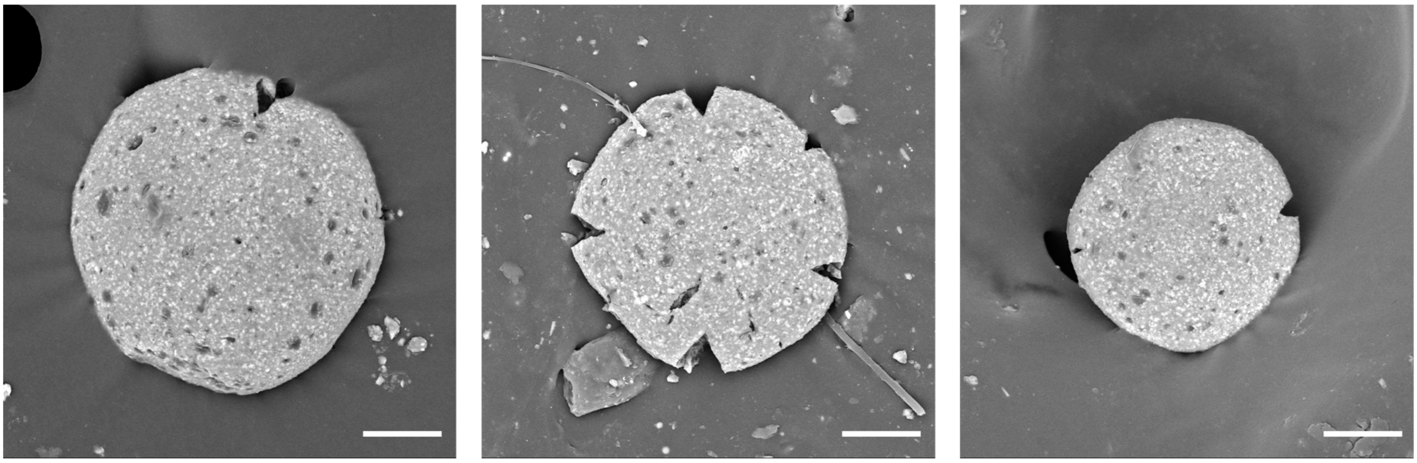

Figure S2. SEM images of broken Na-Alg/Fe_3O_4 microparticles (prepared with $C_{i,Na-Alg}$ at 0.9 mg/mL for the microfluidic experiment). Scale bar 20 μm.

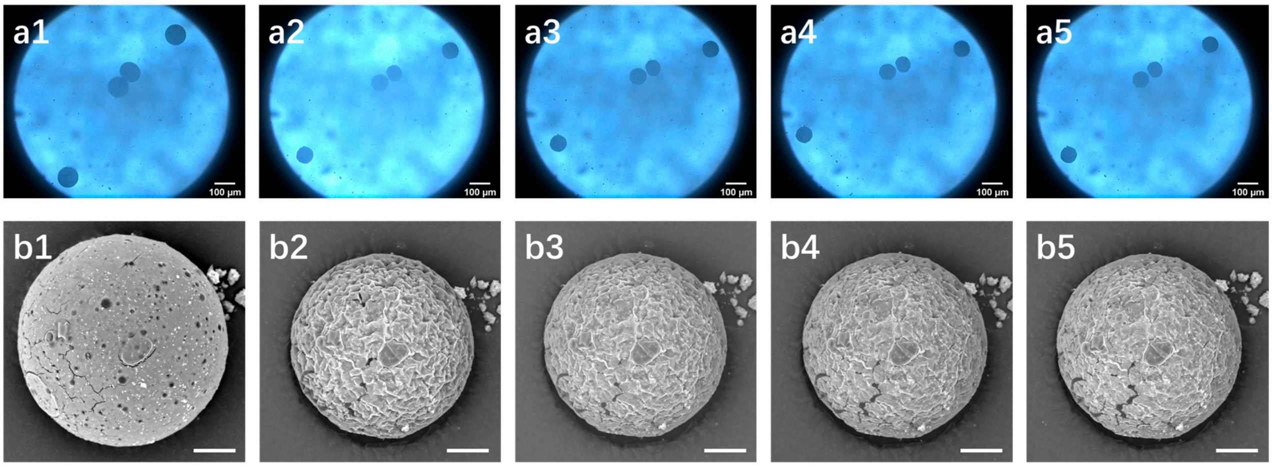

Figure S3. (**a1**–**a5**) Optical microscopic images (in air) and (**b1**–**b5**) SEM images of the same Na-Alg/Fe_3O_4 microparticles (prepared with $C_{i,Na-Alg}$ at 9 mg/mL for the microfluidic experiment). From a1 to a5, also from b1 to b5, the observation time is t0 (just after the preparation), t0+20h, t0+24h, t0+48h, t0+72h. SEM scale bar 20 μm.

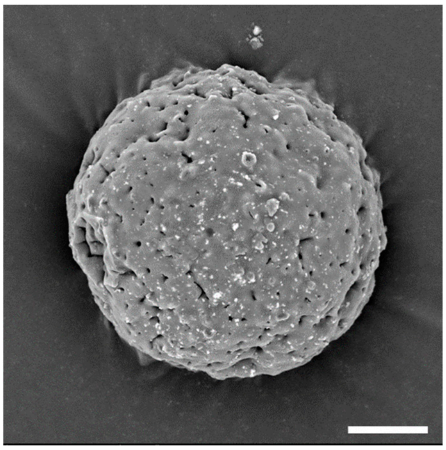

Figure S4. SEM image of a Na-Alg/Fe_3O_4 microparticle stored in air for 72 h after preparation.

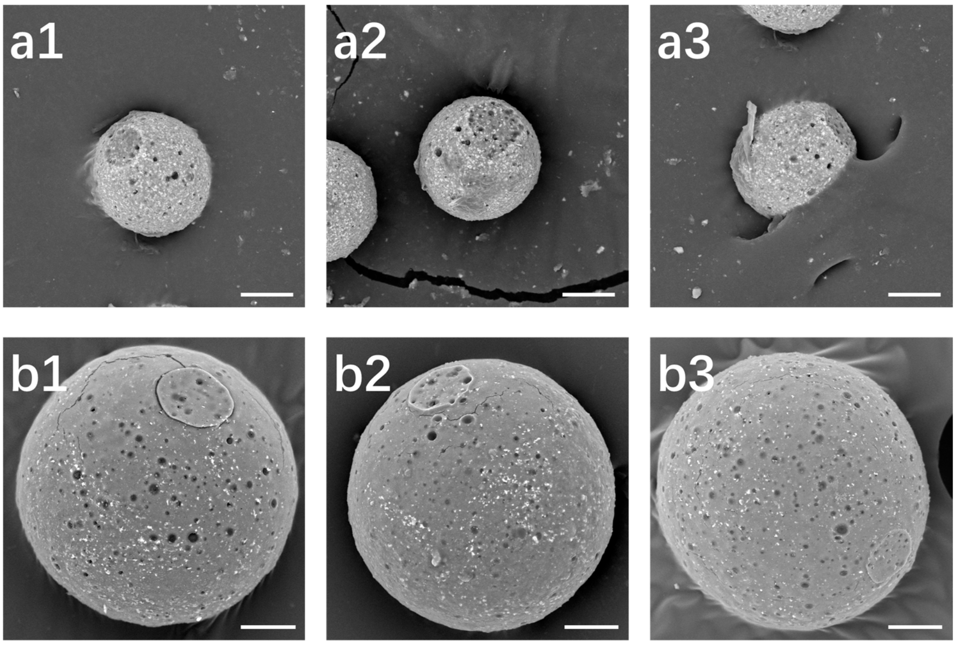

Figure S5. SEM images of Na-Alg/Fe_3O_4 microparticles prepared with $C_{i,Na-Alg}$ at (**a1**–**a3**) 0.9 mg/mL and (**b1**–**b3**) 9 mg/mL for the microfluidic experiment. Scale bar 20 μm.
